# Plant Defense against Insect Herbivores

**DOI:** 10.3390/ijms140510242

**Published:** 2013-05-16

**Authors:** Joel Fürstenberg-Hägg, Mika Zagrobelny, Søren Bak

**Affiliations:** Plant Biochemistry Laboratory and VKR Research Centre ‘Pro-Active Plants’, Department of Plant and Environmental Science, University of Copenhagen, 40 Thorvaldsensvej, Frederiksberg C, Copenhagen DK-1871, Denmark; E-Mails: joelf@life.ku.dk (J.F.-H.); miz@life.ku.dk (M.Z.)

**Keywords:** plant-insect interactions, wound signals, systemic signaling, jasmonates, oligogalacturonic acids, hydrogen peroxide, direct and indirect defense responses, bioactive specialized compounds, digestibility reduction

## Abstract

Plants have been interacting with insects for several hundred million years, leading to complex defense approaches against various insect feeding strategies. Some defenses are constitutive while others are induced, although the insecticidal defense compound or protein classes are often similar. Insect herbivory induce several internal signals from the wounded tissues, including calcium ion fluxes, phosphorylation cascades and systemic- and jasmonate signaling. These are perceived in undamaged tissues, which thereafter reinforce their defense by producing different, mostly low molecular weight, defense compounds. These bioactive specialized plant defense compounds may repel or intoxicate insects, while defense proteins often interfere with their digestion. Volatiles are released upon herbivory to repel herbivores, attract predators or for communication between leaves or plants, and to induce defense responses. Plants also apply morphological features like waxes, trichomes and latices to make the feeding more difficult for the insects. Extrafloral nectar, food bodies and nesting or refuge sites are produced to accommodate and feed the predators of the herbivores. Meanwhile, herbivorous insects have adapted to resist plant defenses, and in some cases even sequester the compounds and reuse them in their own defense. Both plant defense and insect adaptation involve metabolic costs, so most plant-insect interactions reach a stand-off, where both host and herbivore survive although their development is suboptimal.

## 1. Introduction

**Figure 1:**
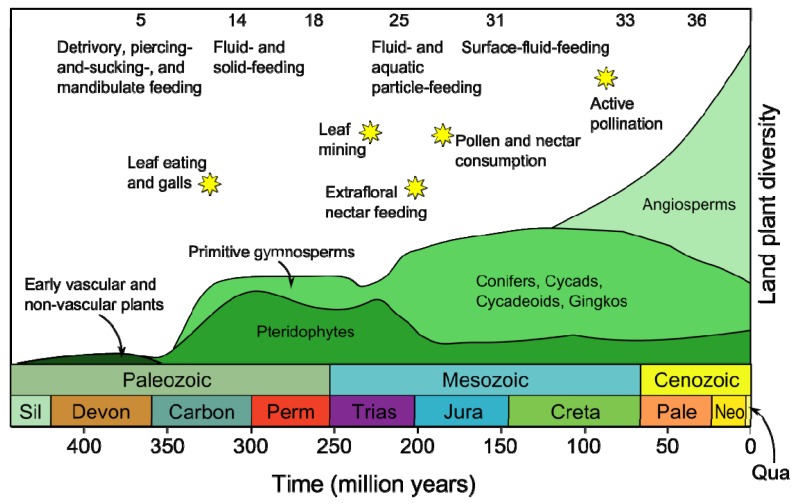
Evolutionary development of plant classes and insect feeding strategies. The evolutionary developments of new classes of plants are shown accompanied by new feeding strategies due to the interactions between plants and insects. The numerals on top refer to the numbers of feeding strategies present. Below are the dominating feeding strategies, followed by the first occurrences of certain feeding modes. The geographic time scale and colors are obtained from the International Commission of Stratigraphy, where Sil—Silurian, Devon—Devonian, Carbon—Carboniferous, Perm—Permian, Tria—Triassic, Jura—Jurassic, Creta—Cretaceous, Pale—Paleogene, Neo—Neogene and Qua—Quarternary (Adapted from [2–4]).

Land plants and insects have coexisted for more than 400 million years. During this time, they have developed refined interactions that affect organisms at all levels, from basic biochemical to population genetics levels. Some of these relationships are mutually beneficial, such as pollination, but most interactions involve insect predation of plants, and plant defense against herbivorous insects. In fact, the predator-host relationship is so common that almost every plant species is eaten by at least one insect species. This has given rise to the co-evolutionary theory [1], which propose that insect feeding on plants has been a determining factor in increasing species diversity in both herbivores and hosts (Figure 1).

The diversity in size and shape of plants varies from only a few millimeters in the microscopic duckweeds (Lemnaceae) to over 100 meters in the enormous Californian redwood trees (*Sequoia sempervirens*). While the lifecycle of some plants last a few weeks, others may live for thousands of years [5]. It is therefore obvious that the strategies employed by plants to defend themselves from the insect herbivores are very diverse. Some species produce traits that affect the insect preference, such as host plant selection and feeding behavior, while some affect their performance, such as growth rate and development. These traits include morphological features for physical defense and the production of compounds for chemical defense.

Insect herbivores have traditionally been divided into generalists (polyphagous) that feed on several hosts from different plant families, or specialists (monophagous and oligophagous), which feed on one or a few plant types from the same family. The generalists tolerate a wide array of defenses present in most plants, while they cannot feed on certain plants that have evolved more unique defense mechanisms. Specialists, on the other hand, use a specific range of host plants releasing defense compounds that at the same time may function as feeding stimulants and provide ovipositioning cues [6–8]. However, this view has recently been challenged [9] since it focuses only on the extremes, while in reality the distribution of insects feeding on one to several plants is a continuum. The paradigm is further based on the fact that feeding generalists and specialists would elicit differential plant responses, which is difficult to prove. It is recommended that such experiments contain at least four species, having the same feeding guild and being in two taxonomic pairs. However, so far no such experiment has been reported [9].

The herbivory defenses of plants may be expressed constitutively or they may be induced and developed only after attack. This is a question of benefit versus cost, since plant defense mechanisms are expensive. Plants are constantly in the dilemma of combining growth and development with defense. This is a problem especially when fitness-limiting resources, like nitrogen, are invested [10] or if the compounds produced are toxic to the plant itself, and not only the herbivores.

This review attempts to cover the whole chain of defense against insect herbivores, from the recognition of a feeding insect, through the production of defense compounds or utilization of physical defenses, to rejection of the plant as food by the insect. Firstly, the early events that induce the defense responses are described, beginning with the interaction in the plant/insect interface. Thereafter, the complex intracellular signaling cascades are treated, with a particular focus on the jasmonate pathway. Finally the different defense responses are explained. The majority of insect herbivores feed on above ground tissues [11], while only 21 root feeding species are known [12]. The main focus in this review will therefore be on plant defense against insect herbivory above ground, with parallels to below ground herbivory whenever possible.

Insect feeding can inflict other pathogens on the plant. The defense against pathogens share several features with the defense against insect attacks, but is beyond the scope of this review, and revised elsewhere [13–19].

## 2. Plant/Insect Interactions Induce Early Signaling

As soon as an insect herbivore starts to feed on a plant, several defense signals are induced, leading to different defense responses. Before describing the signaling mechanism it is however important to point out the ability of the plant to recognize the feeding of an insect herbivore.

### 2.1. Recognition of Insect Herbivore Attack

Plants have the ability to distinguish between herbivory and mechanical damage, such as hail and wind, as well as to recognize ovipositioning. This feature is needed to avoid wasting expensive defense resources, since production and release of defense responses only benefits herbivore-challenged plants.

#### 2.1.1. Feeding Guilds

More than one million herbivorous insect species have been described so far, with different feeding strategies leading to different quantity and quality of mechanical damage on plant tissue. Two thirds of all known herbivores are leaf-eating beetles (Coleoptera) or caterpillars (Lepidoptera) that cause damage with mouthparts evolved for chewing, snipping or tearing [20]. Leaf miners feed on the soft tissue between the epidermal cell layers, while piercing-sucking herbivores, such as spiders and trips, have a tube-like structure used to suck the liquid content from lateral cells. Phloem-suckers such as aphids, whiteflies and other Hemiptera have special stylets that are inserted between the cells and into the phloem. The feeding guilds among root feeding insect herbivores are not as well reviewed as above ground herbivores, but the majority are root-chewers and a few root borers/piercers have also been reported [21].

Plants can evaluate the quality and quantity of leaf tissue damage, a feature studied especially using caterpillars. Caterpillars follow a special pattern when feeding, removing similarly sized pieces of leaf tissue in a highly choreographed and predictable manner. Simulation of repetitive caterpillar wounding by mechanical wounding of *Phaseolus lunatus* (lima bean) resulted in the release of volatiles qualitatively similar to those released by an actual caterpillar attack [22].

#### 2.1.2. Insect Oral Secretions

Plants are also able to recognize compounds in insect oral secretions, which elicit more intense volatile responses than mechanical damage alone [23,24].

**Figure 2:**
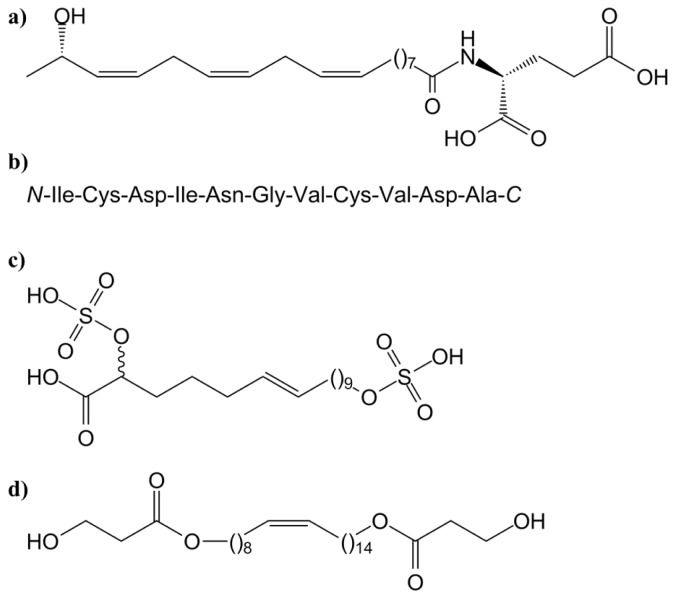
Structures of oral insect secretions. (**a**) Volicitin, *N*-(17-hydroxylinolenoyl)-l-Gln; (**b**) Inceptin, proteolytic peptides of the chloroplastic ATP synthase γ-subunit; (**c**) Caeliferin A16:1, (*E*)*-*2,16 disulfooxy-6-hexadecenoic acid; and (**d**) Bruchin c, (*Z*)-9-tetracosene-1,24-diol bis-(3-hydroxypropanoate)ester.

Conjugation of plant- and herbivore-derived precursors result in the formation of fatty acid-amino acid conjugates (FACs). *N*-17-hydroxylinolenoyl-l-glutamine (volicitin; Figure 2a), first identified in *Spodoptera exigua* (beet armyworm) oral secretions [25], is one of many FACs usually found in oral secretions of Lepidopteran larvae [26,27], such as *Pieris brassicae* (caterpillar of the large cabbage white butterfly) [28]. Volicitin is selectively bound to the plasma membrane, suggesting the existence of a FAC receptor [29]. In *Zea mays* (maize), volicitin activates indole-3-glycerol phosphatase lyase (IGL) that catalyzes the formation of reactive free indoles from indole-3-glycerol [30]. However, some plants, including *Arabidopsis thaliana* (thale cress), *Gossypium hirsutum* (Mexican cotton), *P. lunatus* and *Vigna unguiculata* (cowpea) do not respond to exogenously applied FACs [31].

Other elicitors have been discovered, such as inceptins (Figure 2b), which are disulfide-bonded peptides formed by proteolytic fragments of chloroplastic ATP synthase γ-subunit, produced through the digestion of plant proteins in the gut of *Spodoptera frugiperda* (fall armyworm) [32]. *Phaseolus vulgaris* (common bean), *V. unguiculata* and *Z. mays* respond to inceptin, while *A. thaliana*, *Solanum melongena* (eggplant), *Glycine max* (soybean) and *Nicotiana tabacum* (cultivated tobacco) do not [31,32]. So far, no receptors have been identified for inceptins.

Caeliferins (Figure 2c), disulfoxy fatty acids, were identified in the oral secretions of *Shistocerca americana* (American bird grasshopper) and other grasshopper species [33]. Caeliferins, like FACs, start the release of volatile terpenoids from maize seedlings, but the exact mode of action of these volatiles is not yet known. However, recent successful synthesis of caeliferins makes it possible to further study their function as well as to identify the plant receptors that activate immune responses [34].

Bruchins (Figure 2d), long-chain α,ω-diols, esterified at one or both oxygen atoms with 3-hydroxypropanoic acid, are another class of elicitors, which have been isolated from *Bruchus pisorum* (pea weevil) and *Callosobruchus maculatus* (cowpea weevil) [35]. They are also one of several components found in the oviposition fluids.

Finally, the β-glucosidase in the oral secretion of the larvae of *P. brassicae* elicits the release of volatile organic compounds that attracts the parasitic wasp *Cotesia glomerata* [28].

In contrast to the examples given above, a few elicitors derived from oral secretions actually suppress the defense responses. For instance, salivary glucose oxidase (GOX) secreted by *Helicoverpa zea* (corn earworm) and proteins identified in the salivary glands of *Myzus persicae* (green peach aphid) add up to the oxidative burst and silence the plants defense response, as described in the section on hydrogen peroxide below [36,37]. Furthermore, the proteins from *M. persicae* induced chlorosis and cell death in *Nicotiana benthamiana* [37].

The role of oral secretions in the defense response in roots is still unresolved. Mechanical damage may to be the major cue, since it altered the expression of 80% of the genes responsive to feeding on *Z. mays* by *Diabrotica virgifera* larvae (western corn rootworm) [38]. Compared to leaves, roots are exposed to less abiotic mechanical damage, such as wind, wind-transported particles, rain and heavier animals. It has thus been argued that specific molecular patterns are of less use for recognition in roots, and hence wounding itself is enough to reliably indicate herbivory [39].

#### 2.1.3. Oviposition Fluids

Insect oviposition fluids can give rise to defense responses in the plant as well, making the plant attract egg-eating predators or strengthen its defense in case of a potential future insect herbivore attack [40]. Oviposition by *Diprion pini* (sawfly) on *Pinus sylvestris* (Scots pine) leads to increased production of terpenoid volatiles and decreased ethylene release [41]. Oviposition by *P. brassicae* on *A. thaliana* triggers the expression of defense-related genes as well [42]. However, the chemicals responsible for the defense response have only been identified in *B. pisorum*. Its oviposition fluid contains bruchins that, when added to *Pisum sativum* (pea), elicit tumor-like growths that inhibit the larvae from entering the pod. Furthermore, oviposition of *P. brassicae* on leaves of *Brassica oleracea* (Brussels sprouts) changes the leaf surface chemicals leading to attraction of the egg parasitoid *Trichogramma brassicae* [43].

### 2.2. Early Events in the Plant-Insect Interaction

**Figure 3:**
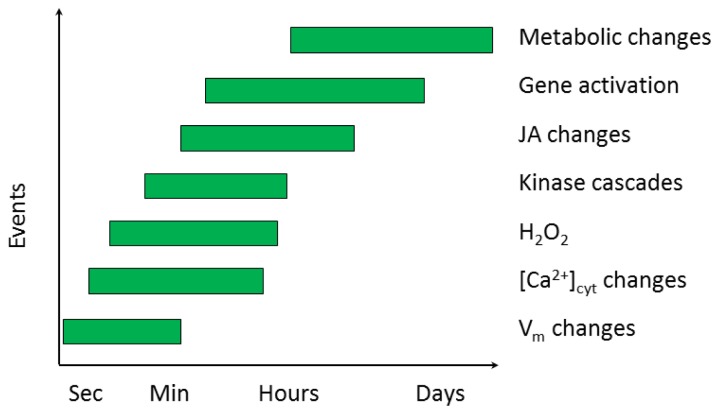
Events in plants after feeding by insect herbivores. Changes in the transmembrane potential (*V*_m_) appear immediately upon herbivory damage and are tightly followed by changes in the intracellular Ca^2+^ concentration and generation of H_2_O_2_. Kinases and the phytohormone jasmonic acid (JA) are detectable within minutes. After roughly 1 h, gene activation followed by metabolic changes is seen (Adapted from [44]).

Most research on plant-insect interaction so far has mainly been focusing on the genomics and proteomics of the late events of plant defense. The early events, recognition and triggering of signal transduction (Figure 3), are on the other hand poorly understood. In this section, the available current knowledge is reviewed.

#### 2.2.1. Electrical Signaling

The plant plasma membrane is in direct contact with the environment, and is therefore able to recognize outer changes and initiate cascade events leading to a possible response. Biotic and abiotic stress will lead to an immediate change in the cell membrane potential (*V*_m_), or modulate the ion flux at the plasma membrane level (Figure 3) [45,46]. The *V*_m_ changes induced by herbivory are followed by a fast electric signal (action potential), which travels through the entire plant from the point where the signal was induced [47]. Although the action potential itself is able to travel at 40 m/s on the cell surface [48], the speed of phloem or xylem transport is no more than 1 cm/min [49]. The *V*_m_ is affected by different signal molecules. One example is the strong depolarizing H_2_O_2_ that may be induced by feeding insects [50]. *V*_m_ depolarization [51] and ion flux [52] have been demonstrated with oral secretions of herbivores, but not with known elicitors alone like volicitin and inceptin. It is nevertheless hypothesized that unknown elicitors affect the activities of various channels and thereby induce electrical signals [49].

#### 2.2.2. Ca^2+^ Homeostasis

Calcium ions function as a second messenger in several plant signaling pathways. In healthy cells, the cytosolic Ca^2+^ concentration is 10,000 times lower than in the apoplastic fluid, and 100,000 times lower compared to the cellular organelles. This creates a driving force for the influx of Ca^2+^ into the cytosol, via channel proteins [53], where it acts as a second messenger [54]. The signal may appear a few seconds after herbivore attack (Figure 3) as a single transient, oscillations, or repeated spikes with specific subcellular localization, lag time, amplitude, and frequency [49]. The signal also differ depending on the organ, tissue or cell type [55]. Following the influx, Ca^2+^ ions are pumped back into the organelles and apoplast via Ca^2+^ pumping ATPases [53].

Calcium fluctuations *in vivo* have been monitored by using fluorescent probes, or the bioluminescence-based aequorin technology [47]. The cell membrane was depolarized upon feeding by Lepidopteran larvae, followed by a transient increase of the cytosolic Ca^2+^ concentration. Furthermore, feeding by *Spodoptera littoralis* (Egyptian cotton worm) on *P. lutanus* and *Ginkgo biloba* (Ginkgo) causes an increase in Ca^2+^. The increase is due to insect oral secretions, and not to mechanical damage [56,57]. Moreover, defense gene transcription is repressed by administration of the Ca^2+^-chelator BAPTA to *P. lunatus* [58].

The Ca^2+^ signal activates calmodulin and other calcium-sensing proteins, such as calmodulin-like proteins, calcineurin B-like proteins, and Ca^2+^-binding protein kinases (CDPKs). This promotes a cascade of downstream effects, like altered protein phosphorylation and gene expression patterns [59,60]. At least two CDPK-signaling pathways seem to exist, one that is involved in crosstalk with mitogen-activated protein kinases (MAPKs), leading to the formation of jasmonates (JAs), and one that is activating defense gene transcription factors independently of the jasmonate and ethylene pathway [61,62]. In addition, the Ca^2+^ concentration in *Medicago truncatula* (barrel clover) is decreased when adding ethephon, a releasing source of ethylene, which indicates ethylene to be a modulator of the Ca^2+^ influx [63].

Except from the Ca^2+^ regulation, little is known so far about other expected ion channels and how they might be regulated by insect-derived elicitors. The same goes for the effector proteins affected by altered Ca^2+^ levels. The focus in the future will be on the characterization of the signal responsive ion channels and the connection to the expected downstream signaling cascades.

#### 2.2.3. Signal Transduction Involving Kinases

Mitogen-activated protein kinase (MAPK) cascades are important pathways downstream of sensors and receptors that regulate cellular responses to both external and endogenous stimuli in eukaryotes, including plants [64–66]. For instance, feeding on *Nicotiana attenuata* (coyote tobacco) by *Manduca sexta* (tobacco hornworm) activates wounding-induced protein kinases (WIPK) and salicylic acid (SA) induced protein kinases (SIPK) in wounded as well as undamaged leaves. The WIPKs and SIPKs in their turn activates other MAPKs, CDPKs and transcription factors [62], and are also critical for the formation of JA induced responses such as methyl-JA (MeJA) and ethylene production after wounding [67], as well as transcription of a gene for ω-3 fatty acid desaturase (FAD7).

## 3. Intracellular Wound Signals

Several different intracellular wound signals have been found, with the phytohormones JA, SA and ethylene being the major players. The JA pathway is induced in response to wounding and tissue-damaging insect feeding, as well as necrotrophic pathogens [68]. SA is known mainly for being activated by biotrophic pathogens, but also by phloem-feeding aphids and spider mites [69], where it acts as a negative modulator through the repression of JA [70,71]. Finally, ethylene is released after attack of insects from several different feeding guilds. It is believed to act in synergy with JA, and has been shown to reduce the production of constitutive defense compounds, while increasing the production of JA and volatiles [72,73]. The focus here will mainly be on the best studied systemic and jasmonate signaling pathways, but downstream events including oligogalacturonic acid and hydrogen peroxide will also be discussed.

### 3.1. Systemic Signaling

In plants attacked by insect herbivores, the expression of several defense genes is induced in undamaged leaves within hours. In Solanaceae this is due to the systemic response, uncovered 40 years ago [74] in studies of the induction of protein inhibitors in *Solanum lycopersicum* (cultured tomato) [75,76]. Systemic signaling has since its discovery served as a model system for studying long-range signaling processes in plants. Several components have been identified that are involved in the systemic induction of defense responses, including systemin peptides derived from larger precursor proteins by proteolytic processing [75,77,78], oligogalacturonides (OGAs) generated by polygalacturonase systemically induced after wounding [79], and jasmonates [80,81].

According to the generally accepted model, systemin trigger the activation of the octa- and hexdecanoid pathways resulting in a burst in production of jasmonate hormones, which leads to the activation of defense genes [82]. Systemin is also involved in Ca^2+^ release from vacuoles, calmodulin synthesis and the opening of ion channels in the plasma membrane leading to depolarization [83]. However, the systemin-encoding gene has not been found in monocotyledons, suggesting that the herbivory response mechanism in dicotyledons is more specific [84].

#### 3.1.1. Systemin and Systemin-Like Peptides

**Figure 4:**
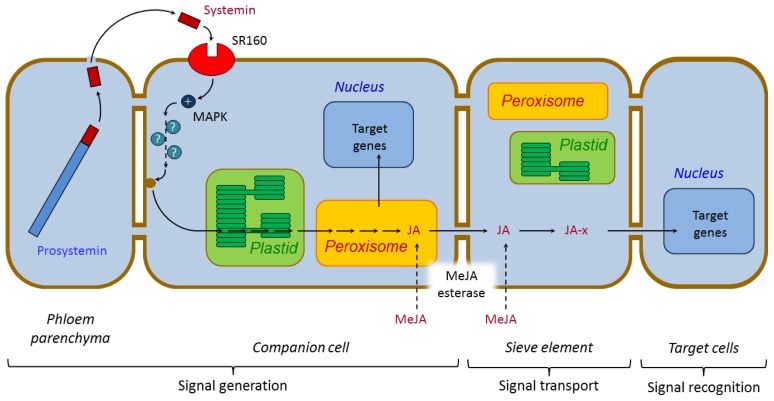
Model of systemic signaling and activation of defense genes in response to wounding by insect attack. After wounding, the systemin peptide is released from the *C*-terminal end of its precursor prosystemin by proteolytic processing. Systemin then enters the apoplast, where it binds to a membrane-bound receptor (SR160) to initiate an intracellular signaling cascade. The cascade includes the activities of a MAP kinase (MAPK), and a couple of unknown intermediates, leading to the release of polyunsaturated fatty acids (PUFAs) by phospholipases, from the membranes. The biosynthesis of JA takes place in the chloroplast and peroxisome within the companion cell, after which it might be transported long distances via the phloem. Plasmodesmatal connections between different cell types are shown as brown pipes. JA or a covalently modified form of JA (JA-x; such as JA-Ile) activates target gene expression in distal undamaged leaves. Esterases may convert exogenous MeJA to JA upon diffusion of MeJA across membranes. For simplicity, cell types presumed to be involved in phloem unloading of the signal are not shown. Mobile signals are shown in red and nonmobile signals in blue. Putative steps are denoted with dashed arrows (Adapted from [83,88]).

The 18-amino acid peptide systemin, found in the vascular bundles of Solanaceae, is proteolytically released from the 200-amino acid precursor prosystemin upon wounding by chewing insects (Figure 4) [85]. However, the proteolytic processing steps, and the enzymes responsible, have not yet been discovered. Though the precursor prosystemin is polar, and contains several proposed cleavage sites, the cleavages that release systemin do not occur in particularly polar regions, or even at conserved sequence motifs (the *N*-terminal cleavage occurs between Leu-Ala, and the *C*-terminal cleavage between Asp-Asn). Prosystemin accumulates in phloem parenchyma cells [86,87], from which systemin is released into the apoplast [88]. Once there, systemin will bind to a 160 kD plasmamembrane-bound receptor (SR160), identified as a member of the leucine-rich repeat (LRR) Ser/Thr receptor kinase family [89]. In the same manner as at a direct wounding site, the binding induces several rapid signaling events, such as membrane depolarization, increased cytosolic Ca^2+^ levels, and activation of a MAPK cascade. The MAPK cascade finally leads to the biosynthesis of JA.

In addition to systemin, systemin-like peptides have been found in *Solanum dulmacara* (nightshade), *Capsicum* spp. (pepper) and *Solanum tuberosum* (common potato), all members of the Solanaceae family [90,91]. Furthermore, functionally related hydroxyproline-rich glycopeptides (HypSys) are found in several of the family members including *Petunia* sp., *S. dulmacara*, *S. tuberosum*, *Nicotiana* spp. and *S. lycopersicum* [92]. Three HypSys have been discovered in the latter (TomHypSys I, II and III), which are all derived from a single polypeptide precursor of 146 amino acids [93]. TobHypSys I and II, found in *Nicotiana* sp., are derived from a 165-amino acid peptide [94]. In both *S. lycopersicum* and *Nicotiana* sp., the genes coding for the precursors are upregulated by MeJA, systemin and wounding, as well as the HypSys peptides themselves [93,94].

#### 3.1.2. Other Signaling Mechanisms

Numerous plasma membrane proteins have been proposed to be acting as wound signal molecule receptors [79,84]. One of them is the β-glucan-elicitor-binding protein (GEBP), isolated from *G. max*, that binds to fungal elicitors [95] resulting in the accumulation of the plant antimicrobial phytoalexins [96–98]. Another type of proteins, containing extracellular leucine-rich regions (LRR), are suggested to be receptors for polypeptide hormones, such as systemin itself [76]. Among these are CLAVATA3 [99], brassinosteroid insensitive (BRI1) [100], phytosulfokines, pollen receptor-like kinase (1 PRK1) [101] and somatic embryogenesis receptor-like kinase (SERK) [102].

The level of abscisic acid (ABA) increases in response to wounding, heat treatment, or application of an electrical current or systemin [103]. Interestingly, ABA has been shown to interact antagonistically to the jasmonate-ethylene signaling pathways, suppressing jasmonate-ethylene-activated transcription [104].

Oxylipins have been proposed to function as signaling molecules and especially the oxylipin traumatin has been suggested as being a trigger of cell division at the wounding site, leading to the development of a protective callus [105].

Finally, a synthetic 12-amino acid peptide derived from a putative extracellular subtilisin-like protease (subtilase) from *G. max* induced defense gene expression when applied to cell culture [106].

### 3.2. Regulation of Defense Responses by Jasmonates

The jasmonate hormones, such as JA, MeJA and JA-Ile, are found in both monocots and dicots. They are involved in several physiological activities, ranging from seed germination, over reproductive development, to senescence. Furthermore, the jasmonates play important roles as signaling molecules in plant defense, particularly against insect herbivores [107].

The jasmonates are rapidly accumulating upon systemic signaling, from an average resting level of about 10–40 ng/g fresh weight in leaves. The increase was observed after 2–5 min in *A. thaliana* and a maximum of at least 40-fold induction was reached within 90 min. Thereafter the JA levels declined to half of its maximum after about 9 h where the levels stayed for at least 24 h [108]. In other plants, such as *S. lycopersicum* and *Nicotiana* spp., the basal levels are reached after a few hours. Compared to leaves, the JA burst in roots is very modest, as seen in *M. truncatula* [109], *N. attenuata* [110] and *Z. mays* [111]. Nonetheless, exogenous application of JA or MeJA to roots induce defense responses, so a higher sensitivity to JA in roots is proposed [39]. In a non-invaded plant, 80% of the JA is in trans-conformation, but immediately after wounding 80% of the JA is found in the biologically more active *cis*-conformation [112]. During the feeding process, a gradient consisting of several oxylipins (including JA) are developing, with the highest concentration directly at the feeding site, and radiating 10–20 mm into the undamaged tissue [113]. The JAs are then transported within the plant and induce the transcription of defense-response genes, both in wounded and unwounded tissues.

#### 3.2.1. Jasmonate Biosynthesis

The JA biosynthesis, mainly studied in *A. thaliana* leaves, takes place in the vascular bundles, as do the synthesis of prosystemin [86,114]. Furthermore, there is a double feedback system where JA biosynthesis is up-regulated by systemin, and prosystemin synthesis is up-regulated by JA.

**Figure 5:**
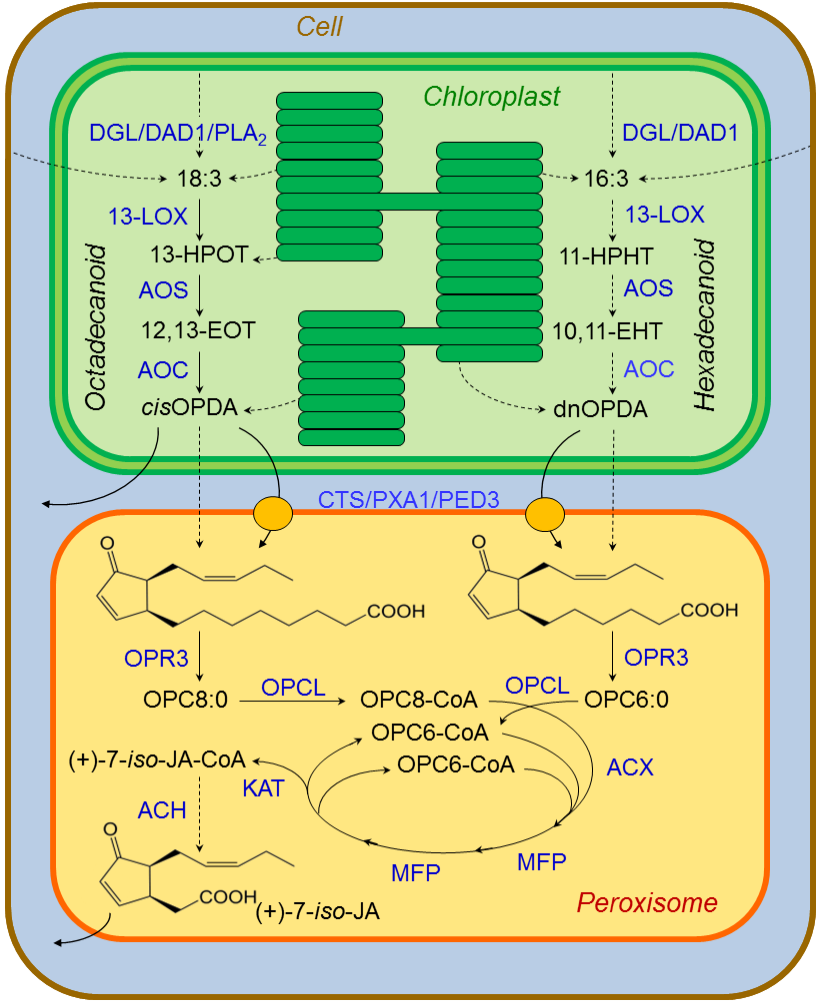
Biosynthesis of jasmonic acid in the chloroplast and peroxisome. Polyunsaturated fatty acids (18:3 and 16:3) released from the cell, chloroplast and/or thylakoid membrane are precursors for the biosynthesis of jasmonic acid (JA). Within the chloroplast, *cis*-(+)-12-oxo-phytodienoic acid (*cis*OPDA) and dinor-OPDA (dnOPDA) are formed via the octa- and hexadecanoid pathways. After transport into the peroxisome, OPDA (dnOPDA) is reduced to OPC8:0 (OPC6:0) and undergoes three (two) cycles of β-oxidation that results in the production of (+)-7-*epi*-jasmonic acid. The reactions are catalyzed by lipoxygenases (LOX), allene oxide synthase (AOS), allene oxide cyclase (AOC), ATP-binding cassette (ABC) transporter COMATOSE (CTS/PXA1/PED3), 12-oxophyto-dienoate reductase (OPR3), OPC CoA ligase1 (OPCL), acyl-thioesterase (ACH), 3-ketoacyl-CoA thiolase [132], acyl-CoA oxidase (ACX) and a multifunctional protein (MFP). Enzymes are shown in blue. Arrows show the well characterized reactions, whereas dashed arrows show steps that are still hypothetical, and for which the corresponding enzymes remain to be identified. (Adapted from [117,133]).

Upon wounding, polyunsaturated fatty acids (PUFAs) [115], and/or fatty acids kept esterified in membrane lipids [116], are liberated from the cell, chloroplast, and/or thylakoid membranes by lipases such as DAD1 (defective in anther dehiscence 1), and DGL (dongle; Figure 5) [117]. Within the chloroplast the PUFAs linolenic acid (18:3), converted from linoleic acid (18:2) by ω-3 fatty acid desaturases (FADs) [118] and/or phospholipase A_2_ (PLA2) [83,119], and hexadecatrieonic acid (16:3), are converted into their hydroperoxide forms through oxygenation by specific lipoxygenases (13-LOXs). The resulting 13-(*S*)-hydroxyperoxy-octadecadi(tri)enoic acid (13-HPOT) and 11-(*S*)-hydroperoxy-hexadeca(tri)enoic acid (11-HPHT) in their turn form a large variety of oxylipins, including JA, through at least six alternative pathways [120–122]. The two JA precursors now follow two parallel pathways; the octadecanoid pathway from 13-HPOT and the hexadecanoid pathway from 11-HPHT [123]. The first step is performed by allene oxide synthases (AOS) that catalyzes dehydrations to form the unstable allene oxides 12,13(*S*)-epoxy-octadecaenoic acid (12,13-EOT) and 10,11(*S*)-epoxy-hexadeca(tri)enoic acid (10,11-EHT). In aqueous media, 12,13-EOT undergoes cyclisation to form *cis*-(+)-12-oxo-phytodienoic acid (*cis*-OPDA), a reaction mediated by allene oxide cyclase (AOC). Four stereoisomers of OPDA may be formed, but only 9*S*,13*S*-OPDA is a precursor for biologically active JA. The 16-carbon homologue dinor-OPDA (dnOPDA) is generated in the parallel pathway from 10,11-EHT [123]. OPDA and dnOPDA are then transported into the peroxisomes, through a mechanism still unresolved. The *Arabidopsis* ATP-binding cassette (ABC) transporter COMATOSE (CTS/PXA1/PED3) has been showed to catalyze the ATP-dependent import of fatty acids into peroxisomes as substrates for β-oxidation [124–126]. Yet, other pathways for dnOPDA must exist, as knockout mutants lack JA-deficiency symptoms (such as male sterility).

Once within the peroxisomes, 9*S*,13*S*-OPDA is reduced by 12-oxophytodienoate reductase (OPR3) to yield 3-oxo-2-(2′(*Z*)-pentenyl)-cyclopentane-1-octanoic acid (OPC-8:0), and dnOPDA is reduced to the corresponding hexanoic acid derivative (OPC-6:0) [127]. OPC-8:0 and OPC-6:0 are then activated through CoA esterification of the carboxylic moiety assisted by OPC-8:0 CoA ligase1 (OPCL1) [128], and a still unknown ligase for OPC-6:0. The hexanoic and octanoic side chains of OPC-8:0 and OPC-6:0 are shortened by two or three rounds of β-oxidation. The β-oxidation involves three core enzymes; acyl-CoA oxidase (ACX), a multifunctional protein (MFP, comprising enoyl-CoA hydratase and β-hydroxy-acyl-CoA dehydrogenase activities) and 3-ketoacyl-CoA thiolase (KAT) forming JA-CoA. The last biosynthetic step is the release of the JA-CoA ester from JA, which is catalyzed by an acyl-thioesterase (ACH), forming the reactive (+)-7-*epi*-jasmonic acid that easily epimerize to the more stable (−)-7-*epi*-jasmonic acid [129,130]. Upon the subsequent transport to the cytoplasm, JA is further modified to methyl-(+)-7-*epi*-jasmonate (Me-7-*epi*-JA) through the assistance of a JA methyl transferase, a (+)-7-*epi*-jasmonyl-l-isoleucine ((+)-7-*epi*-JA-l-Ile) catalyzed by a JA amido synthetase, or other derivatives [131].

#### 3.2.2. Jasmonate Signaling

The JAs are believed to function as long-distance trafficking molecules and be transported in the phloem to undamaged tissues (Figure 2). There are several pieces of evidence for this statement. First, JA biosynthetic enzymes are located in the companion cell-sieve element complex of the vascular bundle [134]. Second, JA has also been found in phloem bundles of *Plantago major* (greater plantain) [134]. Furthermore, the wound-induced systemic responses are improved by the strength of vascular connections between wounded and responding leaves [135]. In addition, the speed of an endogenous signal in tomato plants is estimated to 1–5 cm/h, compared to the rate of phloem transport of 60 cm/h [88].

JA is not the only jasmonate that is supposed to act as a long-distance messenger. JA is considered to be a precursor for JA conjugates that also act as hormones [136]. Strong evidence show that some JA metabolites have unique signaling properties, and that some processes are not controlled by JA, but rather by JA derivatives [137]. For instance, tuberonic acid (12-OH-JA) has long been associated with potato tuber formation and wound healing in tubers [138]. The active signal in defense signaling appears to be the amide-linked isoleucine conjugate JA-Ile rather than JA itself [139,140]. Contradictory to what has earlier been suggested, (+)-7-*epi*-jasmonyl-l-isoleucine ((+)-7-*epi*-JA-l-Ile), and not (−)-JA-l-Ile, is the active form of JA-Ile. Furthermore, the activity was reduced *in vitro* by increasing the temperature, shifting to an alkaline environment, or through methylation [141]. The volatile MeJA, formed by esterification [142], is well studied for its participation in plant-plant communication and extensively reviewed by Cheong and Choi [143]. The details behind the transport of jasmonates and communication between organelles, plant organs and organisms are still unknown.

#### 3.2.3. Jasmonate Regulation of Defense Response

Gene activity is not induced directly by ((+)-7-*epi*-JA-l-Ile itself. Jasmonate ZIM-domain proteins (JAZ) are normally bound to transcription factors, such as MYC2, inhibiting their activity [144,145]. In response to herbivory or mechanical damage, JA will accumulate in the wounded cell and form ((+)-7-*iso*-JA-l-Ile [141]. Biosynthesis of JA-Ile involves the adenylation of JA, followed by the exchange of AMP with isoleucine, and is catalyzed by the amino acid conjugate synthase JAR1 [139,146]. JA-Ile will bind to the coronatin-insensitive 1 (COI1) protein [147], which is the F-box subunit of the E3 ubiquitin ligase of the type Skip/Cullin/Fbox (SCF) protein [132]. Hormone recognition of COI1 favors binding to the JAZ proteins, followed by ubiquitination [148] and guidance to the 26S proteosome for degradation [149]. As soon as the transcription factors are liberated, they will activate gene transcription [148]. The JAZ-proteins are also MYC2-dependent, and will therefore be induced by jasmonates, which explains why the gene activation by jasmonates is only temporary [148].

**Figure 6:**
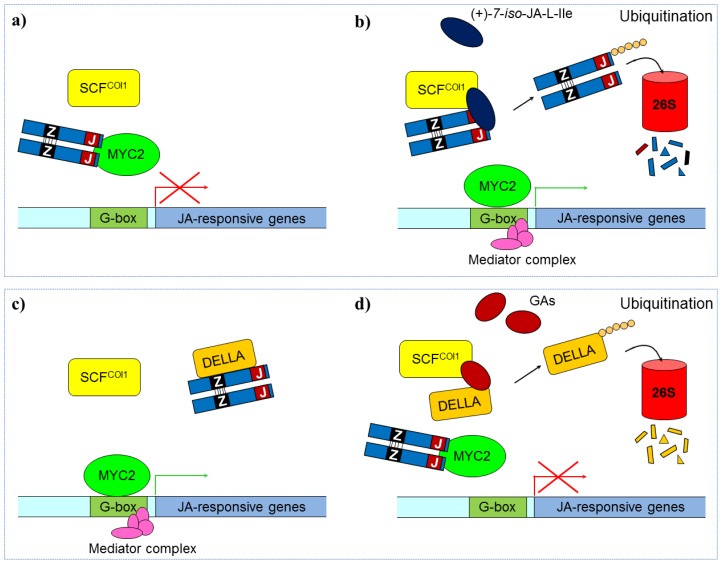
Model of jasmonate regulation of defense responsive genes. (**a**) In the resting state, in the absence of JA, JAZ proteins will bind to transcription factors, such as MYC2, and prevent expression of the JA-responsive genes; (**b**) In the active state, wounding promotes JA biosynthesis, resulting in accumulation of (+)-7-*epi*-jasmonyl-l-isoleucine ((+)-7-*epi*-JA-l-Ile). The hormone will bind to and stabilize the COI1 F-box subunit of the COI1 E3 ubiquitin ligase enzymatic complex (SCF_COI1_), which in its turn bind to the Jas motif (J) of the Jasmonate ZIM-domain protein (JAZ), leading to ubiquitination and subsequent degradation by the 26S proteasome (26S). The transcription factors will now be free to recruit the RNA polymerase II transcriptional machinery to the promoter of the JA-responsive genes, assisted by universal adaptors, such as the Mediator complex; (**c**) In the presence of (+)-7-*epi*-JA-l-Ile and the absence of gibberellic acids (GAs), stabilized DELLA proteins (DELLA) will compete with MYC2 for binding of JAZ, thereby releasing MYC2 for activation of the JA-response; (**d**) If GAs are present, they will bind to DELLA and trigger degradation. This will liberate JAZ, promote the formation of the JAZ-MYC complex, and thereby repress the expression of JA-responsive gene (Adapted from [131,151]).

Furthermore, dominant mutations in the conserved *C*-terminal domain of the JAZ-proteins will stabilize them against further SCF_COI1_-mediated degradation, and reduce the plant’s sensitivity to JA/MeJA [132,148]. In addition, the JA signaling is modulated through the action of gibberellic acids (GAs) [150]. In the presence of JAs and the absence of GAs, DELLA proteins will bind to JAZ1 in *A. thaliana*, and thereby release MYC2 to promote further JA signaling (Figure 6c). If, on the other hand, GAs are present, this will trigger degradation of DELLAs, thereby releasing JAZ1 which will bind to MYC2 and attenuate the JA signaling [151]. JA is also interacting with ethylene [152,153], H_2_O_2_ [154,155], ABA [104] and SA to regulate the expression of downstream target genes in a process extensively reviewed by Lorenzo and Solano [156].

The JAZ protein family is large, ranging from 7 genes in the *Selaginella moellendorffi* lycophyte to 23 in *Z. mays* and is present in at least 13 plant species [157,158]. The 12 *JAZ* genes identified in *N. attenuata* show different expression in roots and shoots [158]. Hence, it is possible that different members of the JAZ protein family may interact with different transcription factors. The interaction of COI1 with JAZ repressors could also be promoted by additional jasmonates, in order to target the JAZ repressors for degradation and thereby release inhibition of jasmonate-responsive genes [129].

### 3.3. Oligogalacturonic Acid

**Figure 7:**
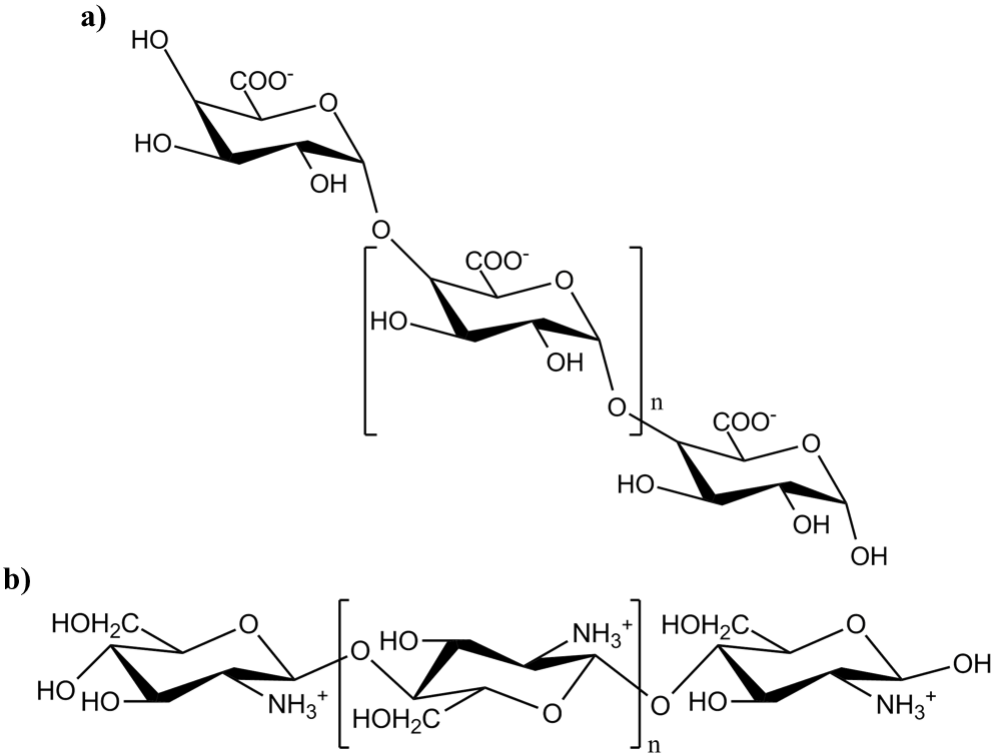
Structures of charged oligosaccharides inducing plant defense-responses. (**a**) The elicitor oligo (α-1,4) galacturonic acid [97] is formed by the action of polygalacturonase (PG) on plant cell wall pectins; and (**b**) chitosan, an oligomer of β-1,4-linked glucosamine, can also act as an elicitor (Adapted from [83]).

The next step in the pathway leading to up-regulation of defense-response genes is the production of oligomeric polymers of galacturonic acid (oligogalacturonides (OGA); Figure 7a). OGAs play several roles in defense, for instance the rapid induction of an oxidative burst [159] through the release of reactive oxygen species (ROS) via a pathway that involves receptor binding [160], activation of a G-protein [161], influx of Ca^2+^ [162,163], stimulation of phospholipase C [164] and induction of several kinases [163].

The OGAs are produced from plant cell walls, through hydrolysis of polygalacturonides, catalyzed by a family of polygalactruronases (PGs) [79] and pectic lyase [165]. The PG gene is activated by JA [166], suggesting that the jasmonate biosynthesis occurs earlier in the signaling pathway.

An issue in understanding the function of PGs is that they can exist both as a single catalytically active subunit and in a complex with the catalytic subunit and a regulatory β-subunit. The β-subunit acts as an inhibitor, as its kinetics is slower than for the catalytic subunit, leading to an 8 h reduction of the activity [79]. Another complication is that PGs are induced by the product of their action, namely MeJA, and would therefore accumulate forever in the absence of any other controls. It has been suggested that the function of the β-subunit is to prevent such a positive feedback loop or that the gene expression and PG action take place in different cellular compartments or in specific cell types [79].

OGA is not the only oligosaccharide that induces defense responses. Oligomers of β-1,4-linked glucosamine (chitosan; Figure 7b) induce the synthesis of proteinase inhibitors in leaves of *S. lycopersicum* [167]. So far, no receptors have been identified for either type of oligomer. However, it is possible that a relatively non-specific interaction takes place, between the charged oligosaccharides and charged membrane lipid components instead of with a specific receptor protein [168]. Oligosaccharides are not mobile within the plant, and are therefore believed to act locally, near the site of production [169], which is directly at the wounding site or in nearby tissues where JA biosynthesis has been stimulated. OGAs can also still amplify defense responses in undamaged tissues, since PG is induced systemically in response to wounding [65].

### 3.4. Hydrogen Peroxide

Herbivory by chewing insects as well as infection by pathogens causes an oxidative burst, characterized by the production of hydrogen peroxide (H_2_O_2_) [170], giving rise to both local and systemic responses [166]. The H_2_O_2_ production has, e.g., been shown to be induced by *H. zea* feeding on *G. max* [171], by *Heterodera glycine* (plant parasitic nematode) feeding on *A. thaliana* [172] and *S. littoralis* feeding on *P. lunatus* [47]. The oxidative burst is further induced by systemin [173] or chitosan [174] in *S. lycopersicum* and by OGA in *G. max* cultures [175]. The oxidative burst is believed to be due to a O_2_-generating NADPH oxidase in the plasma membrane, reviewed by Doke *et al*. [176]. Indeed, inhibition of the NADPH oxidase in *S. lycopersicum* by inhibitors such as diphenylene iodinium (DPI) blocks H_2_O_2_ production and induction of late defense genes coding for proteinase inhibitors. Genes encoding proteins involved in the earlier steps of the signaling pathway (prosystemin, JA biosynthesis, PGs, *etc*.) are not affected [154]. Furthermore, transient expression of a fungal glucose oxidase gene in *S. tuberosum* lead to up-regulation of defense related genes, while the genes from the signaling pathway were unaffected. This proves that H_2_O_2_ is the final signaling molecule in the pathway leading to expression of late defense genes [177,178].

H_2_O_2_ accumulates in or near the vascular bundles and in the intercellular spaces in leaves. The latter location gives rise to the suggestion that H_2_O_2_ acts as a second messenger in stomatal closure induced by OGA [174]. H_2_O_2_ finally diffuses into mesophyll cells, where it up-regulates genes encoding defense proteins, which accumulates in the vacuole [154].

The oxidative burst can, in some cases, be used by the insect itself in order to circumvent the plant defense response. Upon the release of H_2_O_2_ the *V*_m_ will be depolarized, kept constantly reduced for a period of time, and thereby not affected by additional H_2_O_2_. This might be a strategy for the insect to silence the plant defense response, as the plant will then be occupied decreasing the H_2_O_2_ levels using scavenging enzymes such as catalase and ascorbate peroxidase, instead of defending itself against the insect herbivore [47].

Finally, H_2_O_2_ has been shown to activate protein kinases, though it is not clear whether these are involved in the wounding response or belong to signaling pathways leading to defense protein production [179,180].

Nitric oxide (NO) functions as a negative regulator, which reduces the production of wound-induced H_2_O_2_. NO in plant tissues is most likely produced from NO_2_, either non-enzymatically through light-mediated conversion by carotenoids or enzymatically through NADPH nitrate reductases. The role of NO has so far mostly been studied in plant-pathogen interactions, where NO is induced by Jas, and induces the accumulation of SA [181], which then might inhibit the JA biosynthesis [49].

## 4. Defense Responses

Plants have evolved direct defenses such as bioactive specialized compounds (formerly referred to as secondary metabolites or natural products) that could be both inducible and part of the constitutive defense, inducible defense proteins, reallocation of resources from the wounding site to tissues further away, and various morphological features. There are also the indirect defenses, used by the plant to attract, nourish or house predators that can reduce herbivory.

### 4.1. Direct Defense Response

The term “direct defense” is used when plants produce physical barriers against insect herbivores, or compounds that exert repellent, antinutritive or toxic effects on the herbivores themselves. Direct defense mechanisms are described below.

#### 4.1.1. Bioactive Specialized Compounds

Chemical compounds produced by plants have traditionally been divided into primary and secondary metabolites. The primary metabolites are used for growth, development and reproduction. The secondary metabolites, nowadays known as bioactive specialized compounds, are on the other hand used to protect the plant against herbivory and microbial pathogen infection, to attract pollinators and seed-dispersing animals, and as agents in plant-plant competition and plant-microbe symbiosis [119]. Bioactive specialized compounds are targeted especially against biological systems unique to herbivores, such as the nervous, digestive and endocrine organs [182], and are produced both constitutively and upon induction. Bioactive specialized compounds also make a major contribution to the specific odors, tastes and colors of plants [119].

In general, bioactive specialized compounds may act as repellents for generalist insects, and as attractants for specialist insects [183]. Toxic compounds will intoxicate generalist herbivores, while specialists are forced to invest resources in detoxification mechanisms, and their growth and development will therefore slow down [184].

##### 4.1.1.1. Alkaloids

**Figure 8:**
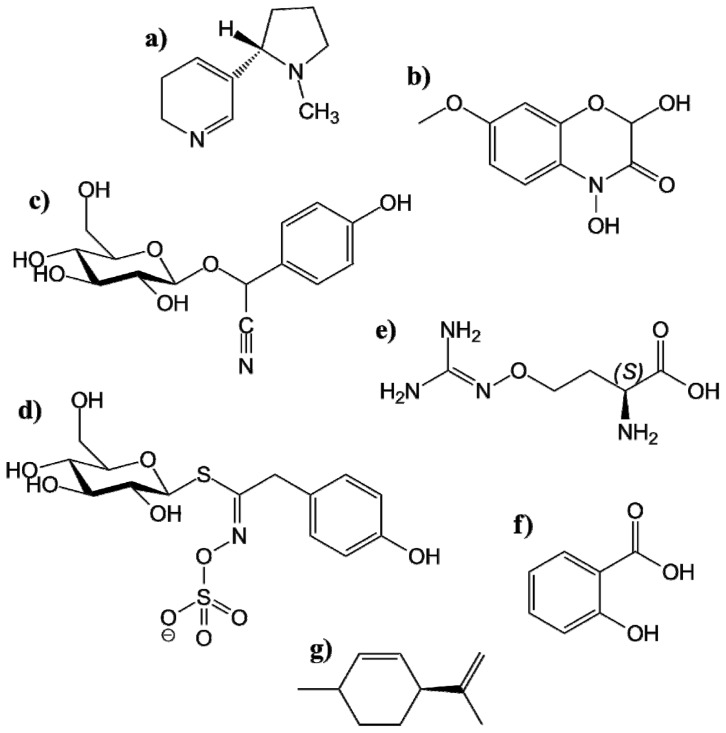
Structures of plant bioactive natural products. (**a**) nicotine, a true alkaloid derived from aspartate and ornithine; (**b**) DIMBOA, a benzoxazinoide derived from indole-3-glycerol phosphate; (**c**) Dhurrin, a cyanogenic glucoside derived from tyrosine, (**d**) Sinalbin, a glucosinolate derived from tyrosine; (**e**) Canavanine, a nonprotein amino acid derived from l-homoserine; **(f**) Salicylic acid, a benzoic acid derived phenol; and (**g**) *S*(−)-limonene, a terpenoid derived from geranyl pyrophosphate [189].

The widely distributed bioactive natural products alkaloids (>15,000 different alkaloids found in 20% of all vascular plants), are prevalently found in the *Leguminosae* spp. (legumes), *Liliaceae* spp. (lilies), *Solanaceae* spp. (nightshade plants) and *Amaryllidaceae* sp. (Amaryllis). They are well known for their metabolic effects in mammals, e.g., caffeine, nicotine, morphine, strychnine and cocaine, and have probably evolved as defense against insect herbivory [59]. The true alkaloids are biosynthesized from amino acids in the roots [185] and accumulated above ground. They are alkaline and contain nitrogen in a heterocyclic ring, as in, e.g., nicotine (Figure 8a) and atropine. The ring structure includes pyridines, pyrroles, indoles, pyrrolidines, isoquinolines and piperidines. The pseudoalkaloids, such as caffeine and solanidine, are alkaline but not derived from amino acids. The protoalkaloids, such as mescaline, are alkaline and derived from amino acids, but the nitrogen is not in a heterocycle [186]. Alkaloids derived from quinolizidine, such as cytisine and sparteine, are efficient feeding deterrents against a number of herbivores [187]. *Solanum demissum* (nightshade potato) containing the alkaloid demissine is resistant to *Leptinotarsa decemlineata* (Colorado beetle) and *Empoasca fabae* (potato leafhopper). On the other hand, *S. tuberosum* contain the sterole derived pseudoalkaloid solanine that the beetles can detoxify, although it has a similar structure as demissine [188].

Pyrrolizidine alkaloids (PAs) are derived from ornithine or arginine and occur naturally in many plants as non-toxic *N*-oxides. However, as soon as they reach the often alkaline digestive tracts of some insect herbivores, they are quickly reduced and forms toxic, uncharged, hydrophobic tertiary alkaloids, which can easily pass through membranes [190]. For instance, in *Festuca arundinacea* (tall fescue) colonized by the fungal endophyte *Acremonium coenophialum*, the feeding of two aphids was reduced because of fungal PAs [191]. Furthermore, PAs were very potent antifeedants and extremely toxic to the aphid *Rhopalosiphum padi* and the wilkweed bug *Oncopeltus fasciatus*. Nevertheless, some herbivores such as *Utetheisea ornatrix* (ornate moth) can detoxify PAs for storage in their bodies and use them in defense against their own predators such as the lacewing *Ceraeochrysa cubana* [192].

##### 4.1.1.2. Benzoxazinoides

*Grammeae* spp., such as maize, rye and wheat, produces the defense-related bioactive specialized compounds 2,4-dihydroxy-1,4-benzoxazin-3-one-glucoside (DIBOA-Glc) and dihydroxy-7-methoxy-1,4-benzoxazin-3-one-glucoside (DIMBOA-Glc, Figure 8b) from indole-3-glycerol phosphate. The conversion is catalyzed by BX1-BX9, of which BX1 cleaves off the glycerol phosphate, BX2-BX5 (cytochrome P450s CYP79C1-4) catalyze the reactions forming DIBOA, BX8/BX9 add the stabilizing glucosyl group, and BX6-BX7 assists in the conversion from DIBOA-Glc to DIMBOA-Glc [193]. A homologue to BX1, indole-3-glycerol phosphatase lyase [194] catalyzes the formation of free indoles in maize, and is activated by volicitin [30]. DIMBOA has been shown to confer resistance to *Ostrinia nubilalis* (first-brood European corn borer), *Helminthosporium turcicum* (northern corn leaf blight), and *Rhophalosiphum maydis* (maize plant louse) [195]. However, DIBOA and DIMBOA may also be used as feeding cues by specialist herbivores. *D. virgifera* is attracted to DIMBOA [196] as well as one of its degradation products MBOA [197]. DIMBOA-Glc may be further converted into 2-β-d-glucopyranosyloxy-4,7-dimethoxy-1,4-benzoxazin-3-one (HDMBOA-Glc) through the action of a JA induced 4-*O*-methyltransferase [198]. Interestingly, HDMBOA that is formed following deglycosylation by a β-glycosidase acts as a strong deterrent to both the generalist *S. frugiperda* and the specialist *S. littoralis* [199].

##### 4.1.1.3. Cyanogenic Glucosides

The cyanogenic glucosides (CNglcs), are found in more than 2600 species from more than 550 genera and 150 families, covering all vascular plant classes including angiosperms, both monocotyledons and dicotyledons, as well as gymnosperms and pteridophytes [194,200,201]. CNglcs are amino acid derived glucosides, originating from aromatic or branched-chain amino acids, such as tyrosine (dhurrin in *Sorghum bicolor*, sorghum [202]; Figure 8c), valine and isoleucine (linamarin and lotaustralin in *Lotus japonicus* (lotus) and *Manihot esculenta* (cassava) [203,204]) and phenylalanine (amygdalin and prunasin in Rosaceae, the rose family, including apples, plums, cherries, peaches and strawberries [205]).

In intact plant tissues, the CNglcs are stored in the vacuole. When the plant tissue is fragmented, for instance due to feeding, the CNglcs are exposed to β-glucosidases located in either the plastids or the apoplast, which leads to hydrolysis and the formation of a sugar and a cyanohydrin that spontaneously decomposes into toxic hydrogen cyanide (HCN) and a ketone or aldehyde. The second step can also be catalyzed by α-hydroxynitrile lyase [206].The volatile HCN is well known for its toxic properties, due to its ability to inhibit the enzyme cytochrome c oxidase in the mitochondrial respiratory pathway [207]. The lethal dose of cyanide for vertebrates, if applied in a single dose, is 1.3–5.5 mg/kg [208] which for instance could be reached by human consumption of 1 kg of white clover [209]. Other roles proposed for CNglcs are as nitrogen storage compounds [210] or as osmoprotectants [211]. The presence of CNglcs in *M. esculenta* tubers increases resistance towards the generalist *Cyrtomenus bergi* (cassava burrower bug) [212]. Furthermore, bitter almond plants containing amygdalin and prunasin are resistant to the larvae of *Capnodis tenebronis* (flatheaded woodborer) [213]. Another example is the larvae of *Hypera postica* (alfalfa weevil), which prefer feeding on the acyanogenic leaves of *Trifolium repens* [214]. With regard to specialist herbivores, on the other hand, CNglcs may act as phagostimulants or oviposition cues. For instance, the larvae of *Zygaena filipendulae* (six-spotted burnet moth) prefer feeding on lotus plants containing cyanogenic glucosides [215] and the larvae of *Spodoptera eridania* (southern armyworm) feed on cyanogenic *P. lunatus* [207]. The larval growth and development is actually retarded in the absence of CNglcs. Furthermore, *Z. filipendulae* is able to both sequester the CNglcs and biosynthesize them *de novo* [216], and uses them for its own defense.

A disadvantage for plants is that the production of CNglcs is expensive, leading to decreased growth and development [217]. The release of HCN in plants may also damage the plant itself. For instance, HCN inhibits the production of phytoalexins, which are used in the defense against microorganisms [218].

##### 4.1.1.4. Glucosinolates

Glucosinolates (GSL) are sulphur- and nitrogen-containing compounds found extensively in *Brassicaceae* and *Capparales*. They are amino acid derived glucosides and at least 120 different structures are known [219]. The GSL are divided into four groups based on the amino acid precursor of the side chain: aliphatic GSL (50%) derived from methionine, indole GSL (10%) synthesized from tryptophan, aromatic GSL (10%) derived from phenylalanine or tyrosine, and structures synthesized from several different amino acids (30%) or with unknown biosynthetic origin. Additional variation is added through chain elongation, oxidation or hydroxylation of the side chain [219]. GSL are more abundant in roots than shoots. Indol-3-ylglucosinolate is most dominant in shoots, while its methoxyderivatives and aromatic 2-phenylethyl GSL is the major GSL in roots. This tissue specificity is believed to be due to difference in volatility, stability in soil and membrane permeability [220]. In roots, the GSL levels are mainly constitutive, while they are inducible in shoots, probably a consequence of difference in selection pressure above and below ground [221].

In a similar fashion to CNglcs, the GSL are located in the vacuole [222] where they are protected from thioglucosidases called myrosinases. Upon tissue disruption, myrosinases will get in contact with GSL and hydrolyze them, resulting in toxic breakdown products, such as isothiocyanates (R–N=C=S), nitriles, and thiocyanates (R–S–C≡N). These breakdown products, usually called “mustard oils”, are responsible for the flavors of several vegetable foods, such as Brassicaceae (mustards, cabbages and radishes) and function both as herbivore toxins and feeding repellents [223]. For instance, GSL prevent *Deroceras reticulatum* (field slug) from feeding on the emerging *Brassica napus* (oilseed rape) cotyledons [224]. Furthermore, the amphipod *Gammarus pseudolimnaeus*, the physid snail *Physella* sp. and limnephilid caddisflies *Hesperphylax designates* and *Limnephilus* sp. preferred senescent *Nasturtium officinale* (watercress) instead of fresh watercress, due to the up to 40 times lower GSL content [225]. In addition, the flee beetle *Phyllotreta cruciferae* feeds preferably on older cotyledons of *Sinapis alba* (white mustard), due to the lower levels of the GSL sinalbin (Figure 8d) [226].

Similarly to the CNglcs, GSL act as repellents of generalists and attractants of specialists. *Psylliodes chrysocephala* (cabbage stem flea beetle) only feed on GSL containing plants [227]. *Ceutorhynchus assimilis* (cabbage seed weevil), use antennal receptors to identify isothiocyanates derived from GSL breakdown, in order to localize and discriminate host plants [228,229]. Other insects inhibit the production of toxic isothiocyanates by redirecting the hydrolysis usually catalyzed by myrosinase toward the formation of less toxic nitriles. This is performed by the specialist *Pieris rapae* (cabbage white butterfly), which utilizes the nitrile specifier protein (NSP) and excrete nitriles in their frass [230]. Instead of a NSP, *Plutella xylostella* (diamondback moth) uses a GSL sulfatase enzyme to convert isothiocyanates and nitriles into desulfoglucosinolates [231]. A study of homozygous lines of *Brassica juncea* (mustard greens), with different myrosinase activity and GSL profiles, gives an example of a plant that uses the glucosinolate system in defense against both generalists and specialists. While the generalist *S. eridania* prefers lines with low GSL concentrations, the specialist *P. xylostella* preferably feeds on lines with low myrosinase activity [232].

The metabolic diversity in toxin production by individual plants can also provide defense against herbivores with different feeding strategies or resistance mechanisms. For instance, breakdown of the nearly 40 different GSL found in *A. thaliana* results in more than 100 breakdown products [59]. Indole GSL that break down in the absence of myrosinase [233] provide a better defense against *Myzus persicae* (green peach aphid) than the more stable aliphatic GSL [234]. More information on GSL can be found in the reviews by Halkier and Gershenzon [235], and Hopkins *et al.* [219].

##### 4.1.1.5. Nonprotein Amino Acids

Many plants, especially Leguminosae produce high concentrations of toxic non-protein amino acids [236]. Both tree and forage legumes contain the arginine analogue canavanine (Figure 8e), which together with its breakdown product canaline is an effective substrates for enzymes utilizing arginine and ornithine. For instance, the arginyl-tRNA synthase of most organisms cannot distinguish between arginine and canavanine, resulting in incorporation of canavanine into proteins, which leads to deleterious effects [237]. However, some insects, such as *Caryedes brasiliensis* (bruchid beetle) and *Sternechus tuberculatus* (curculionid weevil) have an arginyl-tRNA able to distinguish between protein and non-protein amino acids [237].

In contrast to many other bioactive natural products, the non-protein amino acids are not toxic to the plants producing them. For instance, *Canavalia ensiformis* (jack bean) that synthesizes large amounts of canavanine has a protein synthesizing machinery that is able to discriminate between canavanine and arginine, to avoid incorporation of canavanine into its own proteins [119].

Another example is the aromatic amino acid mimosine found in the tropical forage legume *Leucaena leucocephala.* It is usually degraded into toxic dihydroxypyridone (DHP) by ruminant gut bacteria. However, in Central America where *L. leucocephala* is native, the gut bacteria *Synergistes jonesii* is capable of fully metabolizing mimosine and DHP [238,239]. Additional examples are covered in the recent review by Huang *et al*. [240].

##### 4.1.1.6. Phenolics

Plant phenolics include nearly 10,000 individual compounds derived from the shikimic acid and/or malonic acid pathways taking place in the above ground tissues [241]. Among the simple phenolics, derived from phenyl alanine, are simple phenylpropanoids such as caffeic and ferulic acid, phenylpropanoid lactones (known as coumarins) such as umbelliferone and psoralen, and benzoic acid derivatives such as vanillin and salicylic acid. Hydrolyzable tannins are derived from the shikimic acid derived gallic acid. Condensed tannins are derived from anthocyanins that together with other flavonoids such as flavones, flavonols and isoflavones, are the result of condensation of phenyl alanine derived compounds with malonyl CoA. Production of phenolics solely from the malonic acid pathway occurs to some extent in plants, but is more common in fungi and bacteria. The properties of phenolics are very diverse, some are soluble in organic solutions, some are water-soluble carboxyl acids and glycosides, and some, like the condensed tannins, are large insoluble polymers [119].

Phenolics serve as defense compounds by repelling feeding herbivores and inhibiting enzymes, by attracting pollinators and fruit dispersers, by absorbing harmful ultraviolet radiation, as mechanical support in the plant, and by reducing the growth of nearby competing plants [242]. There are a number of examples of phenolics used in defense against insect herbivores. Wheat cultivars containing phenolics are much less attractive to *Rhopalosiphum padi* (cereal aphid) [243]. Light and nutrient stressed *Salix dasyclados* (willow plant), containing three times less phenolics than non-stressed plants, were significantly more attractive to leaf beetle *Galerucella lineola* compared to the controls [244]. Furthermore, benzoic acid derived salicylates in Salix (willow; Figure 8f) leaves halt the growth and development of larvae of *Operophtera brumata* (oak moth) [245]. Leaves of Fragaria (strawberry) contain catechol based phenolics that provide resistance to *Tetranychus urticae* (two-spotted spider mite) [246], because the phenolics covalently bind to the mites digestive enzymes and inactivate them. The cotton phenolic pigment gossypol has repellent effects against numerous insects [247] and is toxic to *Heliothis virescens* (tobacco bollworm), *Heliothis zea* (bollworm) and several other insects [248].

##### 4.1.1.7. Terpenoids

Terpenoids are biosynthesized from acetyl-CoA or glycolytic intermediates. They are classified by the number of isoprene units or five-carbon elements (CH_3_–CH_2_–CH–(H_3_C)_2_); ten-carbon terpenes are called monoterpenes, 15-carbon terpenes are sesquiterpenes, 20-carbon terpenes are diterpenes, 25-carbon terpenes are sesterterpenes, 30-carbon terpenes are triterpenes, 35-carbon terpenes are sesquiterpenes, 40-carbon terpenes are tetraterpenes, and terpenes with even more isoprene units are classified as polyterpenes [119]. Terpenoids are the most metabolically diverse class of plant bioactive natural products (more than 40,000 known structures). Many of them play a role in plant defense, both as components in resin or as volatiles, acting as antifeedants, repellents, toxins or as modifiers of insect development [249].

Many plants contain mixtures of volatile monoterpenes and sesquiterpenes called essential oils, with well-known repellent and toxic effects on insects. Citrus plants produce the terpenoid limonene (Figure 8g), which repels *Atta cephalotes* (leafcutter ant) [250]. Conifers, such as pine and fir, produce monoterpenes that are toxic to several insects, including numerous bark beetle species [251]. The phytoecdysones isolated from *Polypodium vulgare* (common fern) are steroids that disrupt the insect molt because of its similarity to molting hormones [252]. In a similar fashion, some terpenoid amide derivatives can act as insect juvenile hormone analogs [253]. The role of volatile terpenoids in defense against insect herbivores is more thoroughly covered in the later section of this review concerning indirect defenses.

Finally, many terpenoids provide synergistic effects upon release. For instance, binary mixtures of *trans*-anethole and thymol, citronellal, respective α-terpineol, had an almost ten times stronger effect on the mortality rate in *Spodoptera litura* (tobacco cutworm) than would have been the case with simply an additive effect [254].

#### 4.1.2. Digestibility Reduction

Plants produce a number of defense proteins that reduce insect herbivores ability to digest the plant. Anti-digestive proteins limit the rate of enzymatic conversion of ingested food, whereas anti-nutritive proteins limit the utilization of food by altering physical availability and/or chemical identity [255]. The five major classes of defense proteins are: protein inhibitors, α-amylase inhibitors, lectins, chitinases and polyphenol oxidases [84], which are all described below. For a more thorough review, see Bowles [256].

##### 4.1.2.1. α-Amylase Inhibitors

The lectin-like α-amylase inhibitors (α-AI) are found in cereal seeds, such as *Triticum* spp.(wheat) and *Hordeum vulgare* (barley), and in monocots, such as *S. bicolor* and *Z. mays*. The activities of these inhibitors are directed against α-amylases from animals (including insects) and microorganisms, used for starch breakdown, and seldom affect the plant amylases [84]. Wheat α-AIs can inhibit *Tenebrio obscurus* (mealworm), *Tribolium* spp. (flour beetles), *Sitophilus* spp. (wheat weevils) and *Oryzaephilus* spp. (grain beetles) among other stored-grains insect pests, and provide complete protection in transgenic peas towards *Bruchus pisorum* (pea weevil) [257]. α-AI-1 from *P. vulgaris* was tested against 30 agricultural pests, such as insects, mites, gastropods, annelid worms, nematodes, and fungal phytopathogens. It was shown to be a very efficient and selective inhibitor against Coleoptera, Diptera and Hymenoptera, as well as against annelid worms, but not towards Lepidoptera or Hemiptera [258]. Furthermore, transgenic *P. sativum* harboring the cDNA encoding the α-AI of *P. vulgaris* showed protection against various insect herbivores [259,260].

##### 4.1.2.2. Chitinases

Chitin is present in the exoskeleton and peritropic membrane of insects, as well as cell walls of fungi and some algae, but also in nematodes and molluscs [261]. Chitinases have therefore been proposed to have a role in defense against those organisms [262]. Indeed, several transgenic plants overexpressing chitinases have proved to be resistant against insect herbivores. For instance, transgenic *S. lycopersicum* is resistant to *Leptinotarsa decemlineata* (Colorado potato beetle) [263], transgenic *N. tabacum* repels *M. sexta* [264], and *Lacanobia oleracea* (tomato moth) is repelled by transgenic *S. tuberosum* [265].

##### 4.1.2.3. Lectins

Lectins are sugar-binding proteins found especially in storage organs and protective structures of some plants, particularly Leguminosae. The classification of lectins is still evolving because of difficulties with their huge diversity. Today, lectins are divided into six families, based on comparisons of the carbohydrate recognition domains (CRD): legume lectins, cereal lectins, C-, P-, and S-type lectins, as wells as the pentraxins [266], of which only the two first are found in plants.

The glucose/mannose-specific concanavalin A (ConA) from *Canavalia ensiforms* (Jacobean) was the first lectin to be discovered [267]. In addition, ConA expressed in transgenic *S. tuberosum* retarded the development of *L. oleracea* and *M. persicae* by 45% [268]. The lectin *Phaseolus vulgaris agglutinin* (PHA), which is toxic to *C. maculatus* [269], and arcelin, with high sequence similarity to PHA and toxicity towards, e.g., *Zabrotes subfasciatus* (bean weevil) are found in *P. vulgaris* [270]. Furthermore, wheat germ agglutinin (WGA) from corn inhibits *Diabrotica undecimpunctata howardi* (Southern corn rootworm) larval growth by at least 40% [271]. Moreover, WGA inhibits *Lucilia cuprina* (blowfly) larval growth with 50% at a concentration of 2 mM, and induce 100% mortality at 25 mM [272]. Finally, the snowdrop lectin (*Galanthus nivalis* agglutinin, GNA) reduces the development and decreases the fecundity of *Aulacorthum solani* (foxglove aphid) both *in vivo* and in transgenic *S. tuberosum* [273].

When lectins come into contact with the glycoproteins lining the intestinal area of insect herbivores, they are assumed to inhibit the absorption of nutrients [274]. However, the mechanisms of lectins which confer resistance to plants remains poorly understood.

##### 4.1.2.4. Polyphenol Oxidases

Polyphenol oxidase (PPO) enzymes cause the typical browning of plant extracts, mainly fruits, and damaged tissues. This is caused by the spontaneous polymerization and cross-linking of *o*-quinones. PPOs also produce ROS. These two paths occur as soon as the cell compartmentalization is disrupted, and PPO is released from the thylakoid to react with phenolic substrates from the vacuole [275]. PPOs appear frequently upon wounding, and are therefore suggested to play a defensive role. For instance, PPO activity has been associated with resistance to *L. decemlineata* [276], *Melanoplus* spp. (grasshoppers) [277], and Lepidopteran larvae [278]. Furthermore, down-regulation of PPOs in *S. lycopersicum* leaves lead to hypersensitivity to the *Pseudomonas syringae* bacteria, while overexpression increased the disease resistance [279,280]. Moreover, the overexpression decreased the growth rate 2.5-fold in *S. litura*, and increased the mortality up to 3.3-fold [275]. The reason for this is expected to be the highly reactive *o-*quinones, which will covalently modify free amino acids, thereby reducing the nutritive value of proteins [281,282]. PPOs can also be combined with specific phenolic substrates in glandular trichomes to produce a kind of “super glue” to trap smaller insects [84].

##### 4.1.2.5. Proteinase Inhibitors

Four different classes of endopeptidases or proteinases, found in the midgut region of the insect digestive tract, are used by insect herbivores to cleave internal peptide bonds in plant proteins. The most common are the serine proteases, which are found in Coleoptera, Lepidoptera and Orthoptera, which all have neutral or alkaline pH in their midgut lumen content. This class is further divided into the subclasses of trypsin-like, chymotrypsin-like, and elastase-like proteases. The cysteine and aspartic acid proteases have been identified in families with more acidic gut content, such as Coleoptera, Diptera and Hemiptera. The last and by far the smallest class contain the metalloproteinases [283–285]. Phloem-feeding herbivores do not have digestive proteinases and are instead dependent on free amino acids absorbed from the phloem sap as a source of nitrogen nutrients.

Plants have inhibitors for all four classes of proteinases, which can delay larval development without directly causing mortality [286]. They are supposed to inhibit the proteolytic activity of midgut enzymes and thereby decrease the availability of amino acids. This in turn leads to lessening of the synthesis needed for growth, development and reproduction [287]. The inhibitors are often found where the insect attack is most likely to appear, in other words in seeds, bulbs and leaves. In sugarcane, trypsin inhibitors have been detected in leaves, lateral buds and seed tissue, while bi-functional α-amylase-trypsin inhibitors were found in the stem, stem bark, apical meristem and leaf roll. These tissues are the preferential targets for *Diatraea saccharalis* (sugarcane borer) [84].

Insect damage to plant leaves results in an increase of plant inhibitors [288]. Trypsin inhibitors in *G. max* have proved to be toxic against for instance *Tribolum confusum* [289]. Moreover, transgenic tobacco expressing the trypsin inhibitor gene from *V. unguiculata* was resistant to a wide range of insect pests including Coleopterans, such as *Diabrotica* and *Anthonomnous* spp., Lepidopterans, such as *Heliothis* and *Spodoptera* spp., and Orthopterans, such as locusts [290]. Cysteine proteinase inhibitors have been detected in *Ananas comosus* (pineapple), *Grammeae* spp., (barley, maize, rye and wheat), *Oryza sativa* (rice), Soleaceae (potato and tomato) and *V. unguiculata*, with the highest expression in storage organs, like seeds, stem and leaf-root transition zones [84,291].

#### 4.1.3. Reallocation of Resources

To protect valuable resources, they might be reallocated by the plant upon attack. For instance, *Centaurea maculosa* (spotted knapweed) allocates more nitrogen to the shoots upon attack by *Agapeta zoegana* (sulphur knapweed moth) [292]. In this way, the plant can sustain the high photosynthetic activity needed for compensatory growth. Also, feeding on *S. tuberosum* tubers by *Tecia solanivora* (Guatemalan potato moth) larvae led to increased mass of non-attacked potato tubers [293]. Reallocation can also be directed from shoot to root. Oral secretions from *M. sexta* feeding on *N. attenuata* leaves changed the distribution of carbon in favor of the roots [294]. Also, reallocation of starch from *Populus tremuloides* (quaking aspen) leaves to roots was caused by exogenously applying JA to the leaves [295]. Furthermore, application of JA to one half of the *H. vulgare* root system resulted in increased carbon allocation to the non-treated half [296]. The carbon reallocation might be caused by changed invertase activity in roots [297], but the mechanism behind reallocation of nitrogen is still not known [298]. The direction of the transport of resources may be explained by difference in production costs in above and below ground tissues. Acquisition of carbon in photosynthesizing leaves is less costly compared to roots, which on the other hand have ready access to nitrogen in the soil [299].

#### 4.1.4. Morphological Features

To be able to feed, insect herbivores from all feeding guilds will come in contact with the plant surface. Plants have therefore developed a number of physical features such as wax films and crystals, trichomes, leaf and root toughness and quantity, laticifers and resin flow, all described below.

##### 4.1.4.1. Waxes and Crystals

Epicuticular waxes form films and crystals that cover the cuticle of most vascular plants [119]. Aside from their role in desiccation tolerance and protection against pathogens, they also increase the slipperiness, which hinder non-specialized insects from populating the leaf surfaces [300]. The biosynthesis and composition of waxes vary during plant development, and the physical-chemical properties of the cuticle respond on changes in season and temperature [5]. Recently, it was shown that oviposition of *P. brassicae* on *A. thaliana* induce changes in the wax composition, increasing the amount of fatty acid tetratriacontanoic acid (C34), while decreasing the amount of tetracosanoic acid (C24). These changes lead to attraction of the egg parasitoid *T. brassicae* [301].

##### 4.1.4.2. Trichomes

Plant surfaces may further be covered by thorns and spines, for protection mainly against mammals, and trichomes (hairs) against insects [302]. Removal of trichomes results in increased feeding and growth of herbivorous insects [303]. Trichomes have moreover been shown to increase in number in response to insect feeding [5].

Glandular trichomes contain glands that produce volatile or non-volatile bioactive natural products or proteins that repel, deter or poison insects [5]. Non-glandular trichomes, on the other hand, prevent small insects from making contact with the surface, limit their movement or function as entrapment devices. An interesting example of glandular trichomes is seen in *N. attenuata*. Apart from a minor fraction of the highly toxic alkaloid nicotine, the trichomes produce vast amounts of *O*-acyl sugars, which are preferred by the *M. sexta* larvae. This makes the larvae produce volatile branched chain aliphatic acids and thereby attract predators such as *Pogonomyrmex rugosus* (rough harvester ant) [304].

##### 4.1.4.3. Leaf and Root Toughness and Quantity

Leaf toughness interferes with the penetration of plant tissues by mouthparts of piercing-sucking insects and increase mandibular wear in biting-chewing herbivores [305]. For instance, even though mature leaves of *Inga edulis* (ice-cream-bean) are more suitable for growth of fungi, they are avoided by *Atta cephalotes* (fungus-growing ants) due to their toughness [306]. Likewise, mature leaves may be avoided in favor of younger expanding tissues although these contain higher levels of chemical defenses [307]. The cell walls of leaves are also reinforced during feeding [308] through the use of different macromolecules, such as lignin, cellulose, suberin and callose, together with small organic molecules, such as phenolics, and even inorganic silica particles [309].

Roots eaten by insect herbivores exhibit extensive regrowth, both in density, as seen in *T. repens* eaten by *Sitona lepidus* (clover root weevil) [310], and in quantity, as observed in *Medicago sativa* (alfalfa) attacked by clover weevil (*Sitona hispidulus)* [311]. The former might be caused by additional lignification that could increase the toughness of the roots [312]. In addition, genotypes with long fine roots suffered less from herbivory compared to genotypes with short and thick roots [310].

##### 4.1.4.4. Laticifers and Oleoresins

Several plants contain networks of channels in vascular tissues called laticifers and resin ducts. Latex and resins are stored under internal pressure, and when the channels are broken, they are secreted and might entrap or intoxicate the herbivore. Latex laticifers are found in more than 10% of the angiosperms, and is especially common in the tropics [313]. Of the more than 50 latex producing plant families, *Asclepias* (milkweeds) is the one most studied. For instance, the latex of *Cryptostegia grandiflora* (rubber wine) may be transported 70 cm upwards to the wounding site, where it, upon exposure to air, will coagulate and thereby trap small insect larvae [314,315].

Interestingly, many specialist herbivores that feed on latex-producing plants can block the flow of latex from the feeding site by cutting veins or trenches in the leaves [316]. For instance, the milkweed beetles *Labidomera clivicollis*, *Tetraopes melanurus* and *Tetraopes tetrophtalmus* can reduce or even eliminate the flow of latex in *Asclepias* by cutting the leaf veins, and wait until the flow of latex has stopped before feeding [317]. Another example is *Chrysochus auratus* (dogbane beetle) that feeds on *Apocynum cannabinum* (Indian hemp) and opens a channel in the major veins to stop the flow of latex to the margins of the leaves that can then be consumed [318]. Apart from its stickiness, *A. cannabinum* also has toxic or antinutrive properties due to its complex composition of specialized bioactive natural products, such as alkaloids, terpenoids, phenolics and protein inhibitors [319]. In fact, 50–1000 times higher concentrations of these compounds may be stored in the latex compared to the leaf tissues [313].

Conifers produce oleoresins (often termed resin or pitch) that are a mixture of terpenoids and phenolics, and are stored in high pressurized intercellular spaces called ducts. Upon herbivore damage the resin flow will push out stem-boring bark beetles as well as associated pathogens from the bore hole. When the resin is exposed to air, the highly volatile monoterpenes and sesquiterpenes will evaporate, leaving insects trapped in the solidifying resin acids while the wound is sealed [320].

However, specialist insects such as *Scolytus ventralis* (fir engraver beetle) circumvent the defense of *Pinus ponderosa* (Ponderosa pine) by cutting across resin ducts and in that way blocking the transportation of monoterpenes to the feeding site [321]. Moreover, *Dendroctonus ponderosae* (mountain pine beetle) actually uses the resin of *Pinus contorta* (lodgepole pine) as olfactory cue in host recognition and selection [322]. The function of resins in herbivore defense is further reviewed by Trapp and Croteau [251].

### 4.2. Indirect Defense Response

The term “indirect defense” is used when plants attract, nourish or house other organisms to reduce enemy pressure [323]. This is done by producing volatiles, extrafloral nectar, food bodies and nesting or refuge sites.

#### 4.2.1. Volatiles

More than 1000 volatile organic compounds (VOCs), primarily consisting of 6-carbon aldehydes, alcohols, esters and various terpenoids are released from plant flowers, vegetative parts or roots [324,325]. VOCs are used to attract pollinators and predators [24,326] or repel herbivores [327], as well as in communication between or within plants [328–330]. Furthermore, VOCs have been shown to be released from the plant in huge amounts when it is attacked by herbivores [331,332].

Green-leaf volatiles (GLVs) are isomers of hexanol, hexenal or hexenyl acetate with a characteristic odor of freshly mowed pastures. GLVs are immediately released after damage as they are formed from 13-hydroperoxylinolenic acid, which is the first intermediate of the octadecanoid pathway. Other VOCs like methyl salicylate and methyl jasmonates, monoterpenes such as limonene, linalool or ocimene, and sesquiterpens such as bergamotene, carphyllene and farnesene, are usually released within 24 h after attack [324,325,333,334]. Different feeding strategies adopted by herbivores lead to synthesis of different volatiles. For instance, leaf-eaters induce esters, monoterpenes and sesquiterpenes together with JA signaling, while piercing-sucking insect herbivores induce SA-mediated pathways as well [335].

Roots produce different VOCs than leaves. For instance *Z. mays* roots attacked by *D. virgifera* larvae release the sesquiterpene (*E*)-β-caryophyllene as well as small amounts of α-humulene and caryophyllene oxide [336]. Maize leaves, on the other hand, produced over 30 different compounds when exposed to herbivory by *S. littoralis* or the leafhopper *Euscelidius variegatus*. Among those were GLVs, aromatic compounds, homo-, mono- and sesquiterpenes, with (*E*)-β-farnesene and (*E*)-α-bergamotene being the most dominating VOCs of the blend [337]. Also the VOCs released by citrus trees (*Citrus paradisi* × *Poncirus trifoliata*) fed by the root weevil *Diaprepes abbreviates* were different between leaves and roots [338].

Studies have shown that predators associate VOCs, especially terpenoids, with the presence of prey [339,340]. For instance (*E*)-β-farnesene and (*E)*-α-bergamotene released from *Z. mays* attacked by *S. littoralis* attract the female armyworm parasitoid *Cotesia marginiventris* (Cresson) [341]. Transgene expression of the herbivory induced terpene synthase gene *TPS10*, responsible for the formation of these sesquiterpenes in *A. thaliana*, gave the same result [341]. There is also evidence for increased fitness in *N. attenuata* due to predation of the herbivore *M. sexta* by big-eyed bugs (*Geocoris* spp.), which are attracted by VOCs [342]. Moreover, the sesquiterpene (*E*)-β-caryophyllene is released from the roots of European lines of *Z. mays* during attack by *D. virgifera* larvae and attracts *Heterorhabditis megidis* nematodes that feed on the larvae [336]. This attraction has also been studied on *Medetera* fly spp. [343], *Macrolophus caliginosus* (mired bug) [344] and *Scolothrips takahashii* (trips) [345]. In a similar fashion, *C. paradisi* × *P. trifoliata* release terpenes to attract *Steinernema diaprepesi* nematodes, predators of *Diaprepes abbreviates* (root weevil) larvae [346]. Contrary, mechanical wounding of the roots did not induce the attraction in neither *Z. mays* [336] nor citrus trees [346]. On the other hand, specialist insects, such as bark beetles (Coleoptera: Scolytidae), may use the volatile terpenoids from conifers (Gymnospermae: Coniferales and Taxales) as a cue in host recognition [347].

Plants use VOCs to fine-tune their defense according to need, with help from carnivores that use VOCs to distinguish between damaged and undamaged plants, and between plants infested with different herbivore species [348]. One example of this is *N. tabacum* fed on by *H. virescens* larvae, releasing different volatiles during the day and night, in order to attract parasitoids during the day, and repel egg-laying females during the night [24]. Furthermore, when attacked by nicotine-insensitive specialized herbivores, tobacco plants may suppress the induction of nicotine and instead release VOCs [349]. Plants that are attacked are able to communicate with other plants, and alert them of a possible future attack [328,329]. Thereby, the alerted plants will respond stronger once attacked [350]. This communication has for instance been observed between *Artemisia tridentata* (sagebrush) and *N. attenuata* [351] as well as in the hybrid poplar *Populous deltoids* × *Populous nigra* attacked by *Lymantria dispar* (gypsy moth) [352]. This might seem quite strange, as plants usually compete with each other, and this production and release of volatiles would benefit the receiver at the cost of the emitter. One explanation is that the release of VOCs also serves as an internal signal between different parts of the same plant and that the direct vascular connections are restricted [353]. It has also been shown that signaling within the same plant, by using VOCs, is more rapid than the phloem-mediated pathway [351,352]. However, at least in *A. tridenta*, airflow from damaged to undamaged parts is actually necessary for systemic induction [354].

During recent years, more and more field studies of plant/insect interactions are carried out. When the odorous *Melinis minutiflora* (molasses grass) was planted into maize fields, the maize associated herbivory damage decreased. The grass constitutively emits a compound similar to the one released by maize in response to caterpillar damage to attract parasitoids [355]. In another study, the amount of caterpillars in a maize field was decreased by parasitoids, after induction of JA to release VOCs [356]. Moreover, predators were attracted to VOCs associated with beetle-damaged bananas [357] and *P. lunatus* treated repeatedly with JA under field conditions released more VOCs than the controls [358]. They also suffered less herbivory and produced more leaves, flowers and fruits.

#### 4.2.2. Extrafloral Nectar

Extrafloral nectar (EFN) appear in more than 70 plant species spanning angiosperms, gymnosperms and ferns, indicating that it is evolutionary more ancient than floral nectar [359]. In contrast to floral nectar, used to attract pollinators, EFN is secreted on leaves and shoots to attract predators and parasitoids [360–362], but its repellent function has been discussed as well [363]. Examples of crops bearing EFN are *Gossypium herbaceum* (cotton), *Anacardium occidentale* (cashew), *M. esculenta*, *Passiflora* spp. (passion flowers), *Ricinus communis* (castor oil plant), *Prunus* spp. (almond, cherry, peach and plum), and the majority of Leguminosae [364]. EFN consists mainly of sugars (90%), but also amino acids, lipids, proteins, antioxidants, mineral nutrients and bioactive natural products such as alkaloids, phenolics and VOCs [365,366]. However, the compositions vary widely between species, and even between different types of nectars within the same plant species [367]. Although the EFN contains bioactive natural products it is not always toxic [368] and EFN toxic to one insect species might not affect others [369]. The production of EFN is increased by herbivory and decreased in the absence of herbivory [370,371]. EFN secretion is also increased in response to VOCs from herbivore-damaged plants, as showed in *P. lunatus* [372], and by application of exogenous JA onto *Macaranga tanarius* (parasol leaf tree) [373].

The focus in the EFN research field has for long been focused on protective ants, because of their efficient exploratory ability, recruiting strategies and ability to successfully defend their food sources against other players. For instance, leaf damage of *M. tanarius* dramatically increased the rates of EFN secretions, leading to increased numbers of protective ants, and thereafter reduced herbivore pressure [373]. In addition, mites, ladybird beetles, wasps, lacewing larvae and spiders are attracted by EFN released by *Catalpa bignonioides* (southern catalpa), Leguminosae and *Vicia faba* (broad bean) among others, upon herbivory and/or mechanical damage [364].

#### 4.2.3. Food Bodies

Food bodies (FBs) are cellular structures containing mainly carbohydrates, proteins and lipids [374]. They serve as food for ants and are thereby used to attract predators. Because of the high lipid and protein content of FBs, they are considered to be an expensive form of defense. The cost has been estimated to be 2% of the leaf’s construction costs in *Ochroma pyramidale* (balsa tree) [375] and 9% of the above-ground tissue construction cost in *Macaranga bancana* (common mahang tree) [376]. FBs can to some extent be classified as induced defense. For instance, in the tropics the production of FBs in the understory shrub *Piper cenocladum* is tightly connected to the presence of the plants ant *Pheidole bicornis* [377]. The importance of FBs is obvious, since the FBs of *Piper fimbriulatum* growing in Colombia is the main food source for *Pheidole bicornis* ants, which in return efficiently defend the host plant against herbivorous insects, fungi, stem borers and invading vines [378].

#### 4.2.4. Nesting and Refuge Sites

Plants can offer predators like ants, mites and bugs small chambers in the juncture of the midrib and the vein used as nesting or refuge sites (domatia). Ant domatia are restricted to the tropics, while mite and bug leaf domatia can also be found in temperate regions [379]. Removal of leaf domatia will reduce the amount of mites on the flower *Viburnum tinus* [380], while adding domatia to cotton plants will increase the numbers of trips and bugs, leading to improved plant performance [381]. In a similar way to the FBs, domatia is inducible by ants, which was shown on the rain forest tree *Vochysia vismiaefolia* [382].

## 5. Conclusions and Future Perspectives

Since the discovery of systemic signaling in tomato and digestibility-reducing proteinase inhibitors 40 years ago, many courses of action for plant defense against insect herbivores have been identified. Both morphological and chemical defense factors are used to reduce the availability of nutrients. Numerous microarray studies have showed an abundance of genes being induced upon attack by insect herbivores [59]. However, far from all of these identified genes can be associated with the known defense strategies, suggesting that many defense components remain to be discovered. This huge reprogramming of gene expression after herbivory, suggests that herbivory results in a change from growth- and development-oriented to defense-oriented metabolism [383]. There is an interesting evolutionary aspect, as the plant changes the main focus of growth, development and reproduction, in order to defend itself. From an evolutionary point of view, the question arises; at which stage is it more favorable to abandon the defense-oriented metabolism, and switch back to growth- and development-oriented metabolism in order to save what can be saved by reproduction and spreading of seeds?

Despite the last four decades of research in the area, much of plant defense responses against insect herbivores still remain a mystery. Immense effort has been put into the signaling events leading to defense responses. Many components have been discovered, but their order of appearance and how they interact with each other is still unresolved. For instance, the order of the early events, such as calcium flux and phosphorylation cascades, is still poorly understood. Screening for more/new components, mapping of responsible genes and knockout mutant studies are needed to elucidate these pathways and get a more comprehensive view of herbivory defense related signaling events.

The same goes for feedback loops and connections to downstream transcriptional and metabolic changes. The focus has so far been on the jasmonate regulation by JA-Ile, but the interaction between other jasmonates, JAZ-proteins and transcription factors may differ. In addition, it remains to be understood how other wound signals, such as ROS, different phytohormones and insect-derived elicitors interact with the JA-pathway. It is still a mystery how the initial burst of jasmonate production is controlled and how the production is limited in the intact tissues. In fact, the plant defense responses against insect herbivores are shared with other biotic as well as abiotic stress responses, such as changes in transmembrane potential and use of ABA, JA, ROS, *etc*. [384,385]. So how does the plant distinguish between the different sources of stress, and how does it adapt its defense response accordingly?

The topic of volatiles has long been debated, as the concentrations used in laboratories widely exceeds the ones present in nature. The concept is accepted today, but it is still unknown how the insect-derived elicitors are perceived by the plants, as no receptor has been identified. In addition, plants respond differently to elicitors. For instance, maize reacts very strongly to applied elicitors, whereas *Arabidopsis* and cowpea are affected only by single elicitors, and others such as tomato are non-responsive [31]. What is the reason for this? Is it related to their geographical origins and corresponding selecting agents? Could it be a consequence of the long-running domestication of maize [386]? The emergence of next generation sequencing techniques together with more powerful and cost efficient metabolite profiling instruments makes screening across a wider spectrum of plant species possible which might be able to shed some light on these questions.

Although present in both monocots and dicots, most of the current understanding of the JA pathway comes from studies of the dicots *Arabidopsis*, tobacco and tomato. However, studies on monocots have revealed interesting contrasts. There is a tendency of more *JAZ* genes being present in monocots than dicots. For instance, maize contains 23 JAZ proteins, which is nearly twice as many as tobacco [157,158]. This suggests involvement in other hormone signaling pathways or abiotic stress tolerance [387]. Furthermore, the NAC transcription factor RIM1, a negative regulator of JA biosynthesis in rice, has not yet been identified in *Arabidopsis* and may thus be specific to monocots [387,388]. Finally, systemin and systemin-like peptides are found only within Solanaceae and are absent in monocots. Without systemin, how can the JA burst be initiated?

Overall, there is a lack of studies comparing the defense responses between different plant species. So far, studies have mostly been carried out on model organisms, such as ants and Spodoptera larvae, feeding on crop or model plants like *Arabidopsis*, maize, rice, tomato and tobacco. Some research has been carried out on trees, such as poplar and eucalyptus, although most focus has been on laticifers and oleoresins. Not all plants are expected to respond the same way to insect herbivory. It would therefore be of interest to see more diversity among the plants and insect herbivores studied. This would probably result in new interesting insights and a much wider view of plant defense responses against insect herbivory.

Generally, insect herbivory have been considered to increase towards the tropics and decrease with increased altitudes [389]. However, this view is likely to be inaccurate. Most studies of herbivory along environmental gradients are biased towards point herbivory, measuring percentage of leaf damage by a single leaf chewing insect species on North American and European plant species [390]. Conclusions drawn thereof cannot simply be extrapolated onto other geographical areas. For instance, due to different climates, North American and European plants are, in contrast to plants on the southern hemisphere, mostly deciduous. Thus up to 50% of leaf nitrogen and phosphorous are transferred back into the plant seasonally [391], which, while being beneficial for phloem-sucking insects, reduces herbivory of chewing insects. Obviously more studies are needed, and they should cover other feeding styles, more insect and plant species, and more geographical locations with different climates instead of varying latitudes or altitudes. Most important though, the same methods should be applied at all sites. When doing so the traditional view on altitudes, latitudes, temperatures and their effect on herbivory may be challenged [392,393].

Although plant defense against insect herbivores has mostly focused on above ground herbivory, some features specific to below ground herbivory are emerging. Since below ground tissues are not exposed to stress to the same extent as above ground tissues, their perception of insect herbivory is not as specific. Mechanical damage may be the major factor triggering the defense responses [38]. Also, different signals seem to be used below ground, indicated by attenuation of the JA burst in roots. Still, below ground tissues are responsive to JA, implying a higher sensitivity for JA in roots. Furthermore, the *JAZ* genes show different tissue expression [158], which may be due to different roles in the signaling. There are many cases were the defense compounds biosynthesized in roots differs from the ones produced in leaves. The complexity of the volatiles identified also tends to be higher in leaves than roots. One reason could be that volatiles cannot easily diffuse through the soil and reach potential herbivores or predators, nor be used for inter- and intraspecific communication, and thus are not as suitable in roots as they are in leaves. Instead, reallocation of resources and compensatory growth is a more apparent defense strategy in roots. Finally, induced defense responses seem less common in roots, while they are evident in leaves of several plants. It has been argued that inducible defenses in roots provide little benefit to the plant [39]. Since many root herbivores are specialists, they would probably have gained resistance towards inducible defenses anyway.

From a biotechnological, food-developmental, and breeding point of view, understanding the defense systems of plants and learning how to apply the knowledge is of course of huge interest. For instance, modifications of the JA pathway has been proposed [394]. However, due to the extensive crosstalk with other hormone signaling pathways, increased resistance against one certain insect herbivore might result in susceptibility towards another. Furthermore, some defense responses might have negative effects on the environment and humanity as well, as they involve toxic bioactive natural products and proteins reducing digestibility of plant material. Still, reducing the need for synthetic insecticides, by developing crop plants resistant to insect herbivores, would be of significant gain for the food and production industry, both at an economical and environmental level.

Plant defense against insect herbivores is just one of multiple layers of interactions. In addition, plants are being utilized as nutrition source and shelter by parasitizing fungi, bacteria and viruses, along with vertebrates such as birds, lizards and mammals, as well as other invertebrates like worms and snails. Together with plants, these players are involved in complex interaction networks. To elucidate these fascinating interactions both biochemical and ecological studies, and combinations thereof, are required.
